# The pattern and correlates of intimate partner violence among women in Kano, Nigeria

**DOI:** 10.4102/phcfm.v8i1.1209

**Published:** 2016-11-29

**Authors:** Tanko S. Tanimu, Stephen Yohanna, Suleiman Y. Omeiza

**Affiliations:** 1 Department of Family Medicine, Aminu Kano Teaching Hospital, Nigeria; 2 Bingham University Teaching Hospital, Jos, Nigeria; 3 National Orthopedic Hospital, Dala, Nigeria

## Abstract

**Background:**

Intimate partner violence (IPV) has been increasingly recognised as a major public health and human rights problem that cuts across all populations, irrespective of social, economic, religious or cultural groups.

**Objectives:**

The objectives of this study were to determine the prevalence, pattern and correlates of IPV among women attending the General Out Patient Clinic of Aminu Kano Teaching Hospital, Kano, Nigeria. It was also designed to determine the pattern of health complications associated with IPV as well as the perception of women on intimate partner violence.

**Methods:**

This was a cross-sectional, hospital-based study. Three hundred and ninety-three women aged 15–49 years who were in or had ever been in an intimate relationship were recruited. An interviewer-administered questionnaire was used to collect data about their socio-demographic characteristics while information on IPV was obtained using the Composite Abuse Scale. The data were analysed using the Statistical Package for Social Science (SPSS) version 16.0.

**Results:**

The prevalence of IPV within the previous year was 42.0%. Of all the 393 participants recruited in the study, 46.6% had experienced emotional/psychological violence, harassment/controlling behaviour was present in 43.3%, physical violence was reported in 29.0%, sexual violence was present in 21.9% and 37.9% of the participants had experienced severe combined abuse. Being married (*χ*^2^ = 24.726, *p* = 0.000) and pregnancy reduced the risk of IPV (*χ*^2^ = 6.690, *p* = 0.030), while polygamous family setting (*χ*^2^ = 9.734, *p* = 0.008) and an extended family type (*χ*^2^ = 9.593, *p* = 0.023) were associated with an increased risk of IPV. Alcohol consumption by the partner (*p* = 0.000, OR 2.335, CI 1.151–3.230) was found to be a positive correlate as well as a complication of IPV. Other patterns of health complications that were significantly associated with IPV were depression (*p* = 0.000, OR 3.517, CI 4.061–22.306), miscarriage (*p* = 0.004, OR 2.080, CI 1.591–2.269) and the presence of physical injuries in the participants (*p* = 0.024, OR 2.405, CI 2.345–4.234). One hundred and fifty-nine (40.5%) of the participants agreed that a husband is justified for beating or hitting his wife and neglecting the child was the reason given by most of the participants (26.7%) to justify IPV.

**Conclusion:**

The high prevalence of IPV among women of reproductive age in this study shows that it is an important problem that women would rather not talk about or have accepted as a norm. It is associated with poor physical and mental health of women who are victims.

**Recommendation:**

It is therefore recommended that physicians routinely screen for IPV especially in patients with depressive symptoms, miscarriage and physical injuries. Screening will be a safe and cost-effective means for identifying women experiencing IPV, leading to appropriate interventions that will decrease further exposure to IPV and its adverse health consequences.

## Introduction

Intimate partner violence (IPV) has been defined as behaviours within an intimate relationship that cause physical, psychological or sexual harm to those in the relationship, including acts of physical aggression, sexual coercion, psychological abuse and controlling behaviours.^1^ For the purpose of this study, intimate partners include current spouses; current non-marital spouses (boyfriends/girlfriends); former marital partners (divorced spouses, separated spouses); and former, non-marital spouses (boyfriends). Intimate partners may be cohabiting, but need not be.^2^ Violence against women is the most pervasive and yet under-recognised human rights violation in the world. It is also a profound health problem that saps women’s energy, compromises their physical and mental health, erodes their self-esteem and prevents them from achieving their full potential.^3^

In 48 population-based surveys from around the world, between 10% and 69% of women reported being physically assaulted by an intimate male partner at some point in their lives, and that 15% – 30% had been assaulted in the previous year.^3,4^ A cross-sectional study by Zungu et al. 2010 on IPV among women attending a public hospital in Botswana showed the lifetime and past-year prevalence of IPV to be 49.7% and 21.2% respectively.^5^

In Nigeria, studies on the prevalence of IPV have reported a wide range of values. A cross-sectional study by Envuladu et al. in Jos,^6^ the northern part of the country, reported a prevalence of 31.8% among pregnant women, while Iliyasu et al. in Kano found a prevalence of 58.8% among female university students in Northern Nigeria.^7^ In a cross-sectional study carried out among a sample of women attending a primary health centre in Ile-Ife, south-western Nigeria, the prevalence of IPV in the previous 12 months was found to be 36.7%.^8^ A similar study on IPV among women of childbearing age in a primary health care centre in eastern Nigeria, reported a prevalence of 46.3% for the previous 12 months.^9^

IPV is not simply a matter of family privacy, individual choice or inevitable facet of life. It is a complex problem related to patterns of thought and behaviour that are shaped by a multitude of forces within families and communities.^1^

In Nigeria, some women justify domestic violence perpetrated by husbands depending on the situation. In the 2003 Nigerian Demographic Health Survey, 64.5% of women and 61.3% of men were reported to support wife beating in at least one of the six scenarios described in the survey.^10^ There is a paucity of data on the pattern and extent of IPV in the region.

## Materials and methods

This was a cross-sectional, descriptive, hospital-based study carried out between 1st August and 30th September, 2015, at the General Out-Patient Clinic (GOPC) of the Family Medicine Department of Aminu Kano Teaching Hospital (AKTH), Kano, Nigeria. The aim and objectives of the study were read to eligible participants, whence a voluntary, written informed consent was obtained from each participant.

The study population consisted of all women aged 15–49 years who were in or had ever been in an intimate relationship, presenting to the GOPC of AKTH.

Through a systematic, random sampling 393 women were recruited for this study.^8^

Sample size was calculated using the formula:^11^

*n* = *Z*2 *p q* /*d*^2^        [Eqn 1]

where *n* = the desired minimum sample size (when population is greater than 10 000), *Z* = the standard normal deviation, set at 1.96 which corresponds to 95% confidence interval, *p* = prevalence of IPV among women attending a primary health centre in Ile-Ife = 36.7%,^8^ *q* = 1 – *p* = 0.633, *d* = level of precision usually set at 5% = 0.05.

Therefore, *n* = (1.96)^2^ × 0.367 × 0.633/(0.05)^2^ = (1.96)^2^ × 0.2323/0.0025 = 0.8924/0.0025 = 356.96 ≈ 357 participants.

Ten per cent of the calculated minimum sample size (36 women) was added to account for attrition, missing or incomplete data. Thus, the number of women to be recruited for this study will be 357 + 36 = 393 participants.

Participants were included if they consented and were aged 15–49 years, currently married, divorced, or involved in an intimate relationship. Women with known psychiatric illness were excluded because their concentration, memory and judgement could be impaired.

A pre-tested, structured questionnaire was administered by the investigator to eligible women after obtaining informed consent from individual participants. Information on IPV was obtained using the Composite Abuse Scale (CAS).^12^ The CAS consists of 30 items presented in a six-point format requiring respondents to answer ‘never’, ‘only once’, ‘several times’, ‘monthly’, ‘weekly’ or ‘daily’ in a 12-month period. A preliminary cut-off score of 7 divides respondents into abused and non-abused. Preliminary recommended cut-off scores for the individual subscales are as follows: Physical Abuse (I), Emotional Abuse (3), Harassment (2), Sexual abuse (1) and Severe Combined Abuse (I). Information on the perception of women on IPV was assessed using extracts from the 2003 Nigeria Demographic and Health Survey model questionnaire which has a number of questions about the perception of women on intimate partner violence.^10^ The Patient Health Questionnaire-2 (PHQ-2) was used to screen for depression among the respondents.

All data collected were analysed using Statistical Package for Social Science (SPSS) version 16.0 software. The Chi square test was used to assess the associations of demographic variables with type of violence. Logistic regression analysis was used to assess the correlates of intimate partner violence. A *p*-value of less than or equal to 0.05 was considered statistically significant.

### Ethical clearance

Ethical clearance for this study was sought and obtained from the Research Ethics Committee of AKTH, Kano, Nigeria.

## Results

The age range of the study participants was between 16 and 45 years. The mean age was 27 (SD ± 6.29) years and the modal age group was 25–34 years (47.3%).

Other socio-demographic characteristics of the study participants are as shown in Table 1.

**TABLE 1:** Socio-demographic characteristics of the participants recruited in the study (*N* = 393).

Characteristics	Frequency (*N*)	%
Age group(in years)		
15–24	141	35.9
25–34	186	47.3
35–44	63	16.0
45 and above	3	0.8
Marital status		
Single	65	16.5
Married	305	77.6
Divorced	20	5.1
Separated	3	0.8
Religion		
Islam	287	73.0
Christianity	103	26.2
Traditional	3	0.8
Ethnicity		
Hausa	223	56.7
Fulani	36	9.2
Yoruba	37	9.4
Igbo	26	6.6
Others	71	18.1
Pregnancy status		
Currently pregnant	81	20.6
Not pregnant	308	78.4
Don’t know	4	1.0
Family setting		
Monogamy	238	60.6
Polygamy	92	23.4
Not applicable	63	16.0
Occupation of participants		
Civil servant	68	17.3
Unemployed	155	39.4
Trader	75	19.2
Student	54	13.7
Others	41	10.4
Participants’ level of education		
None	3	0.8
Non-formal	24	6.1
Primary	36	9.2
Secondary	173	44.0
Tertiary	157	39.9

Of all the respondents, 165 (42.0%) had experienced a form of violence in the preceding 12 months while 228 (58.0%) had not experienced IPV in any form.

Harassment/controlling behaviour was the most common form of violence experienced by 71 (43.3%) of the participants. Forty-eight (29.0%) had experienced physical violence, 36 (21.9%) had experienced sexual violence while 10 (5.8%) had experienced emotional/psychological violence. Sixty-three (38.2%) participants had experienced severe combined abuse.

The age of the participants (*χ*^2^ = 3.693, *p* = 0.254), ethnicity (*χ*^2^ = 5.916, *p* = 0.205), religion (*χ*^2^ = 0.99, *p* = 0.844), occupation (*χ*^2^ = 5.261, *p* = 0.265) and level of education (*χ*^2^ = 6.364, *p* = 0.150) were not significantly associated with IPV. However, the correlates found to be significantly associated with IPV on bivariate analysis were:

Marital status: IPV was significantly associated with marital status (*χ*^2^ = 24.726, *p* = 0.000). Being married reduced the risk of IPV while divorced women were more likely to have experienced IPV.

Pregnancy status: There was an association between pregnancy status and IPV on bivariate analysis (*χ*^2^ = 6.690, *p* = 0.030). However, on further analysis, there was no significant independent association (*p* = 0.678, OR 1.188, CI 0.527–2.676).

Type of family setting: The polygamous family setting was associated with increased risk of IPV (*χ*^2^ = 9.734, *p* = 0.008).

Type of family: Extended family setting was associated with increased risk of IPV (*χ*^2^ =9.593, *p* = 0.023).

The pattern of health complications associated with IPV were as shown below:

Physical injury: There was an increased risk of sustaining physical injury following assault from an intimate partner. This association was found to be statistically significant (*χ*^2^ = 84.658, *p* = 0.000).

Miscarriage: Miscarriage was significantly associated with IPV (*χ*^2^ = 68.465, *p* = 0.000). Women were at an increased risk of having a miscarriage following assault from their partners.

Substance use by the respondents: There was an association between the increased use of substances such as sleeping pills and IPV (*χ*^2^ = 38.305, *p* = 0.000).

Alcohol use by the partner: Alcohol consumption by the partner was significantly associated with IPV (*χ*^2^ = 17.494, *p* = 0.000).

Depression: IPV was significantly associated with depression in women (*χ*^2^ = 41.163, *p* = 0.000).

Body mass index: Overweight and obesity were associated with IPV (*χ*^2^ = 27.639, *p* = 0.000). Being overweight or obese increased the risk of IPV.

Blood pressure: IPV is significantly associated with the development of hypertension in women (*χ*^2^ = 33.394, *p* = 0.000).

The variables that were significantly associated with IPV on bivariate analysis were further subjected to multiple logistic regression analysis to adjust for the effect of confounders. The independent correlates of IPV identified are presented in Table 2.

**TABLE 2:** Multiple logistic regression analysis of correlates of intimate partner violence.

Variables	Odds ratio	95% Confidence interval	*p*
Miscarriage	2.080	1.591–2.143	0.004*
Alcohol use by partner	2.3355	1.1513.230	0.000*
Depression	3.517	4.061–22.306	0.000*
Physical examination findings	2.4048	2.345–4.234	0.024*

* statistically significant.

**FIGURE 1:**
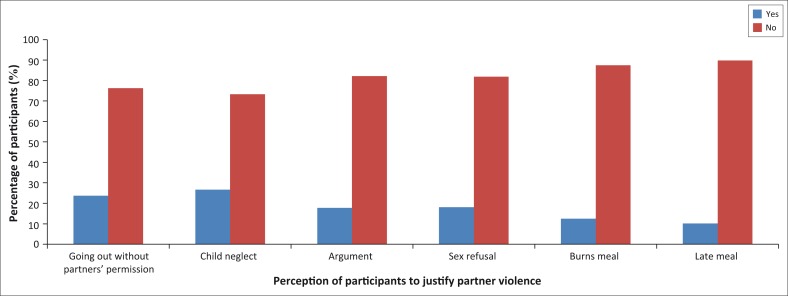
The perception of participants to justify intimate partner violence.

One hundred and fifty-nine (40.5%) of the participants agreed that a husband is justified for beating or hitting his wife for at least one of the reasons asked. Neglecting the child was the reason given by most of the participants, 105 (26.7%), to justify wife beating while cooking meals late was the reason given by the least, 40 (10.2%), participants to justify wife beating. Two hundred and thirty-four (59.5%) of the participants did not think IPV was justified for any reason. The perception of participants on IPV is summarised in Figure 1:

## Discussion

Health professionals are often the earliest point of contact for survivors of violence. Family physicians, through effective case management, co-ordinated and continuing care, can alleviate some of the negative effects of partner violence on women suffering in silence. In addition, through publications of research findings, family physicians can create awareness of the burden of the problem and as such promote advocacy for women empowerment.

The prevalence of IPV within the previous year in this study was 42.0% which is within the range of global estimates of IPV prevalence of between 20% and 50%.^1^ It is also similar to the prevalence of 45.6% in Africa.^3^ In Nigeria, a similar prevalence of 46.3% was reported by Ilika and colleagues in a clinic-based study among the Igbo community in Neni, Anambra State.^9^ Okenwa and colleagues reported a prevalence of 29% among women of childbearing age in Lagos, South west of Nigeria.^13^ The lower prevalence in their study could be due to the differences in methodology and the socio-cultural characteristics of the study populations. The prevalence of IPV reported in this study could still be underestimated because of beliefs that issues concerning families and intimate relationships should not be discussed as it is seen as a ‘private matter’.^14^

The present study measured four different patterns of IPV among all the participants (393) involved in the study.

Physical violence: In the present study, 29.0% of the 393 participants experienced physical violence from their intimate partners. The result from this study is comparable with reports of physical violence by Iliyasu et al. in Kano (27.5%), and Gyuse and Ushie in Jos (26.5%).^7,15^ It is fairly higher than the reports of physical violence from Eastern Nigeria by Ilika et al. in Anambra State (15.8%) and Okemgbo et al. in Imo State (20.1%).^14,16^ These differences could be due to differences in cultural characteristics of the study populations. Northern Nigeria has a distinct culture and tradition of female seclusion (‘*Purdah*’), practice of polygamy, forced marriages and desire for large families. In the Igbo tribe, it is culturally unacceptable to beat a pregnant or lactating mother.^14^ Based on comparison on studies about the association between pregnancy and IPV across the world, many studies reported that the association remains uncertain whether, in fact, the risk of IPV initiation or escalation increases, decreases or remains the same as a result of pregnancy. The reason that was advanced was that studies that examined the risk of IPV during pregnancy did not examine the patterns of IPV around the period of pregnancy through comparisons of IPV prevalence rates in the pre-pregnancy, pregnancy and post-pregnancy periods.^6,8,9,15,17,18^ IPV against women before pregnancy was a strong risk factor for abuse during pregnancy and after delivery, and abuse during a previous period was a strong indicator of subsequent abuse.^17,18^

Emotional/psychological violence: In this study, 46.6% of the 393 participants experienced emotional violence which is comparable to the prevalence of 47.5% reported by Al-Nsour in Jordan and 50.8% reported by Iliyasu et al. among female university students in northern Nigeria.^7,19^ Although psychological violence was found to be the most prevalent form of IPV in the present study, it is still much higher than the 23.0% reported in a study by Okenwa and colleagues among women of childbearing age in Lagos, Nigeria, in which psychological violence was also the commonest form of IPV.^13^ Zungu et al. admitted the difficulty in measurement of psychological IPV due to diversity in cultures.^5^

Controlling behaviour/harassment: In this study, 43.3% of the 393 participants experienced controlling behaviour from their intimate partners. A study by Adebayo et al., in Lagos, Nigeria, found a higher prevalence (71.9%) among their study participants.^20^ This wide range suggests a variation in the degree to which such behaviour is normative or acceptable in different cultures.

Sexual violence: In this study, 21.9% of the 393 participants experienced sexual violence which is within the range of 10% and 50% reported from the WHO Multicountry study.^1^ The result is also consistent with prevalence rates reported in Imo state (21.3%), Kano (22.2%) and Lagos (21.0%).^19,20,22^ The prevalence in this study is higher than the prevalence reported from a study in Jos (10.7%), which was conducted among an obstetric population.^15^

The differences in age, religion, ethnicity and educational level were not statistically significant in the present study. This may be related to the fact that IPV cuts across age, class, race, ethnicity, religion and national boundries.^1,3,4^ This may also be due to the patriarchal nature of Nigerian ethnic groups and religions.

This study revealed that a high percentage of the participants (40.5%) agreed that a husband is justified for hitting or beating his wife under the conditions examined in this study. The high level of support expressed for wife beating in this study confirms that violence against women is accepted as a cultural norm. A respondent from a focused group discussion by Ilika et al. among Igbo women of Ozubulu community in Anambra State was of the view that ‘a woman deserves beating if she talked back on her husband, refused him sex, presented a poorly cooked food, or late meal, and failed to care for the children’.^14^ Another respondent in a focused group discussion by Odimegwu et al. among the Tiv in Nigeria was of the opinion that ‘if you are not yet beaten by your husband, then you do not know the joy of marriage and that means you are not yet married’.^21^ Thus, the perception of women to justify wife beating varies according to cultural norms and personal attributes, which suggests that designing an effective intervention to eliminate violence against women would require culturally acceptable programmes.

This study revealed a high percentage (67.9%) of the victims of IPV sustained physical injuries such as bruises, red eyes, lacerations, etc, following assault from their partner. This is an expected finding given the high prevalence of IPV found in this study.

This study has found a significant positive association between IPV, depression and substance use among the victims of abuse. This finding is consistent with reports from similar studies within and outside Nigeria.^1,4^ IPV is an established risk factor for depression and depression has also been described as a health consequence of IPV.^1^ Thus, IPV can be conceptualised as a risk factor, correlate or outcome of substance use and depression. IPV being a significant cause of psychiatric illness in women, its exclusion in this study would tend to underestimate the prevalence that was reported. However, women with known psychiatric illness were excluded because their concentration, memory and judgement could be impaired, thus information obtained cannot be relied upon. Its exclusion is a limitation for this study.

This study found that women are at an increased risk of having a miscarriage following assault from their partners (p = 0.004, OR 2.080, CI 1.591–2.143). This finding is consistent with the reports from the studies that showed that women with a history of physical or sexual abuse are also at an increased risk for unintended pregnancies, sexually transmitted diseases and miscarriages.1,^6^,^9^ Therefore, targeting IPV prevention interventions to pregnant women in prenatal care settings may reduce health care costs associated with adverse outcomes in pregnancy.

## Conclusion

The high prevalence of IPV among women of reproductive age in this study shows that it is an important problem that women would rather not talk about or have accepted as a norm. It is associated with poor physical and mental health of women who are victims.

This study provides insight for policy makers, physicians and researchers who are attempting to tackle the global issue of IPV.

### Recommendation

It is therefore recommended that physicians routinely screen for IPV especially in patients with depressive symptoms, non-specific complaints, miscarriage and physical injuries on the face, trunk and upper limbs. Screening will be a safe and cost-effective means for identifying women experiencing IPV, leading to appropriate interventions that will decrease further exposure to IPV and its adverse health consequences.
